# Simulation of Gamma-Ray Attenuation in Zeolite–Polymer Composites for Low-Cost Sustainable Radiation Shielding

**DOI:** 10.3390/polym17233141

**Published:** 2025-11-26

**Authors:** Ahmed Alharbi, Hamed Alnagran, Saleh Alashrah

**Affiliations:** Department of Physics, College of Science, Qassim University, Buraydah 51452, Qassim, Saudi Arabia; hnjran@qu.edu.sa (H.A.); ashrh@qu.edu.sa (S.A.)

**Keywords:** zeolite, polymer composites, radiation shielding, photon attenuation, sustainable materials

## Abstract

Lightweight and lead-free radiation shields are increasingly developed to overcome the toxicity and handling challenges associated with conventional heavy-metal-based materials. In this study, the γ-ray attenuation behavior of polymer–zeolite composites was examined by reinforcing high-density polyethylene (HDPE) and polylactic acid (PLA) with natural clinoptilolite zeolite at concentrations of 10–40 wt%. Photon-interaction parameters, including the linear attenuation coefficient (μ), half-value layer (HVL), mean free path (λ), and effective atomic number ($Z_{\mathrm{eff}}$), were evaluated over 15 keV–15 MeV using the Phy-X/PSD platform. Zeolite incorporation consistently enhanced photon attenuation, particularly at low energies dominated by the photoelectric effect. At 15 keV, the HVL decreased from 0.60 cm to 0.08 cm for HDPE and from 0.043 cm to 0.033 cm for PLA as the zeolite loading increased to 40 wt%. Correspondingly, $Z_{\mathrm{eff}}$ increased from 2.7 to 4.3 for HDPE and from 6.5 to 11.6 for PLA, while μ reached approximately 41 cm^−1^ and 56 cm^−1^ at 15 keV for the respective 40 wt% composites. Beyond about 1 MeV, differences between compositions became minimal as Compton scattering dominated. PLA–zeolite composites exhibited higher μ and lower HVL than HDPE–zeolite, whereas HDPE maintained an advantage in mixed-field environments owing to its hydrogen-rich matrix. The results confirm that zeolite-reinforced polymers are safe, low-cost, and lightweight materials suitable for radiation shielding in medical, nuclear, and aerospace applications.

## 1. Introduction

Radiation shielding is a cornerstone of radiation safety in healthcare, nuclear engineering, and industrial applications, serving to protect humans and equipment from harmful ionizing radiation. The demand for effective shielding materials is particularly high in medical imaging and radiotherapy facilities, nuclear reactors, waste storage systems, and non-destructive testing environments. Traditional shielding materials such as lead, tungsten, and concrete have long been favored due to their high density and strong photon attenuation capabilities [1,2,3]. However, these materials present significant drawbacks. Lead is toxic, brittle, and environmentally hazardous, while concrete is heavy, prone to cracking, and difficult to recycle or reuse. These limitations have motivated extensive research toward developing alternative materials that are lighter, safer, and more sustainable.

Polymers have emerged as promising matrices for radiation-shielding composites owing to their low cost, ease of fabrication, and design flexibility. Materials such as high-density polyethylene (HDPE) and polylactic acid (PLA) are widely used in radiation-shielding studies as reference polymers, where they serve as convenient model matrices for evaluating the influence of different fillers on photon attenuation performance [4,5,6,7,8,9,10,11]. Although HDPE and PLA are not considered radiation-resistant polymers for long-term structural applications, they are commonly employed as baseline systems in computational studies because they allow the effect of filler composition, loading fraction, and photon-interaction physics to be isolated without the complexity of advanced high-performance polymers. For applications exposed to high accumulated radiation doses, the same zeolite formulations can in principle be transferred to more radiation-resistant aromatic or conjugated polymers such as polyimide [12,13]. In the present study, HDPE and PLA are therefore used strictly as model matrices for benchmarking photon attenuation and are not proposed as long-term structural shielding materials.

To overcome the intrinsic limitations of neat polymers, numerous studies have investigated composites enhanced with high-atomic-number (high-*Z*) fillers such as bismuth oxide (Bi_2_O_3_), tungsten trioxide (WO_3_), lead oxide (PbO), and barium sulfate (BaSO_4_). Adding these fillers enhances photon attenuation properties and reduces the half-value layer by increasing the probability of photoelectric and Compton interactions [14,15,16]. However, the widespread use of heavy-metal fillers presents new challenges, such as increased material weight and potential toxicity during both processing and disposal. As a result, there is growing interest in developing environmentally friendly, low-density, and cost-effective alternatives that deliver acceptable photon shielding without relying solely on toxic or very-high-density phases.

Among the potential candidates, natural zeolites, which are microporous aluminosilicate minerals, have emerged as a promising yet comparatively underexplored option for this purpose. They are naturally abundant, chemically stable, and inexpensive, making them attractive for sustainable radiation shielding applications. Zeolites are environmentally benign and can be readily incorporated into polymer matrices to produce lightweight composites with improved radiation attenuation [17,18,19,20]. Several studies have shown that zeolites possess mass attenuation coefficients (μ/ρ) comparable to those of clays and soils and only slightly lower than those of conventional concrete, confirming their potential as sustainable shielding fillers. Further investigations have also demonstrated the successful integration of natural zeolites into polymeric and cementitious materials, achieving enhanced γ-ray attenuation while maintaining good environmental performance [19,20].

In this context, zeolite–polymer composites are proposed as sustainable, lightweight alternatives to conventional heavy-metal-based shielding systems. This work investigates how zeolite loading (10–40 wt%) modifies the photon-interaction behavior of HDPE and PLA matrices and benchmarks their performance against standard shielding materials. Using the Phy-X/PSD platform, photon attenuation metrics such as the mass and linear attenuation coefficients, half-value layer (HVL), mean free path (MFP), and effective atomic number ($Z_{\mathrm{eff}}$) are computed over 15 keV–15 MeV and cross-checked against a dedicated GEANT4 Monte Carlo transmission model. Finally, the simulated performance of the zeolite–polymer composites is compared with representative lead-, steel-, and concrete-based shields reported in the literature to clarify the niche where such lightweight, lead-free systems can offer a meaningful balance between attenuation efficiency, mass, and environmental impact.

## 2. Theory

### Photon Attenuation

The attenuation of a monoenergetic photon beam in matter follows the exponential law    

(1) $$I=I_{0}e^{-\mu x},$$

where $I_{0}$ is the incident intensity, I(x) is the transmitted intensity after traversing a thickness *x*, and μ is the linear attenuation coefficient [21]. The mass attenuation coefficient relates attenuation to material density ρ via

(2) $$\mu_{m}=\frac{\mu }{\rho }.$$

A convenient shielding metric is the half-value layer (HVL)—the thickness required to reduce the intensity by 50% [22]:

(3) $$\mathrm{HVL}=\frac{\ln2}{\mu }.$$

For compounds and mixtures (e.g., zeolite–polymer composites), the total atomic cross-section $\sigma_{t,a}$ can be evaluated from the elemental mass fractions $w_{i}$ and atomic weights $A_{i}$ using [23]:

(4) $$\sigma_{t,a}=\mu_{m}N_{A}\sum_{i}\frac{w_{i}}{A_{i}},$$

with $N_{A}$ Avogadro’s number. The total electronic cross-section $\sigma_{t,el}$ is obtained from mixture relations that account for the atomic numbers $Z_{i}$ and atomic abundances $f_{i}$ of each constituent element [24,25]. Two composite descriptors are defined:

(5) $$Z_{\mathrm{eff}}=\frac{\sigma_{t,a}}{\sigma_{t,el}},\;N_{\mathrm{eff}}=\frac{\mu_{m}}{\sigma_{t,el}}.$$

For heterogeneous materials such as polymer–zeolite composites, all photon-interaction parameters were evaluated using the standard mixture rule, in which the mass attenuation coefficient of the composite is expressed as

(6) $${\left(\frac{\mu }{\rho }\right)}_{\mathrm{mix}}=\sum_{i}w_{i}{\left(\frac{\mu }{\rho }\right)}_{i},$$

where $w_{i}$ is the mass fraction of the *i*-th constituent element or oxide.

## 3. Materials

The oxide composition of the zeolite used in this study was taken from Gili and Hila [20], whose measurements provide representative mass fractions of SiO_2_, Al_2_O_3_, K_2_O, Na_2_O, CaO, and MgO for natural clinoptilolite. Similar oxide compositions for natural zeolites have been reported in other studies [18,19]. Table 1 summarizes the oxide fractions used in this work. These oxide fractions were subsequently converted into elemental mass fractions for input into both Phy-X/PSD and GEANT4. Because the work was carried out entirely through simulations, no laboratory materials or equipment were used, and therefore supplier details are not relevant.

The computational modeling approach used high-density polyethylene (HDPE) as the foundational polymer matrix, reinforced with clinoptilolite zeolite at loadings of 10–40 wt%. Two reference systems were included for baseline comparison: pure HDPE (100 wt%) and pure zeolite (100 wt%). Composite densities were estimated using the standard mixture rule for multiphase systems [26]:

(7) $$\frac{1}{\rho_{\mathrm{composite}}}=\sum_{i}\frac{\omega_{i}}{\rho_{i}},$$

where $\rho_{\mathrm{composite}}$ is the density of the mixture, $\omega_{i}$ is the weight fraction of component *i*, and $\rho_{i}$ is the density of component *i*. In this study, $\rho_{\mathrm{HDPE}}=0.94$ g cm^−3^, $\rho_{\mathrm{PLA}}=1.25$ g cm^−3^, and $\rho_{\mathrm{zeolite}}=2.20$ g cm^−3^. Table 2 lists the densities used in GEANT4 for the investigated HDPE–zeolite and PLA–zeolite systems.

## 4. Methods

### 4.1. Phy-X/PSD Computations

Radiation-shielding metrics were obtained with Phy-X/PSD 2020, which aggregates photon interaction data primarily from the NIST XCOM database to ensure consistency with experimental standards [27]. The code returned the linear attenuation coefficient (μ), mass attenuation coefficient (μ/ρ), half-value layer (HVL), mean free path (MFP), effective atomic number ($Z_{\mathrm{eff}}$), equivalent atomic number ($Z_{\mathrm{eq}}$), and exposure/energy absorption buildup factors across the studied energies. Inputs comprised the elemental mass percentages of each HDPE–zeolite formulation together with their mixture-rule densities (Table 2). Phy-X/PSD assumes homogeneous materials and monoenergetic, narrow-beam irradiation, making it suitable for baseline, benchmarkable predictions of γ-ray attenuation prior to transport simulation.

### 4.2. GEANT4 Monte Carlo Simulations

**Figure 1:**
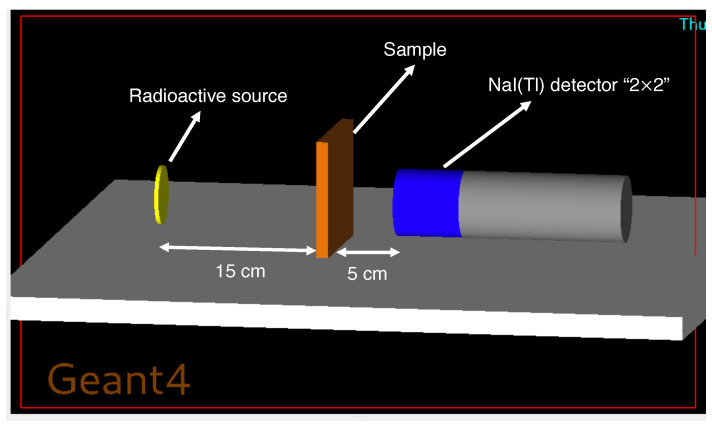
GEANT4 attenuation setup (schematic). A γ source (yellow) irradiates the HDPE–zeolite slab (orange); a NaI(Tl) detector (2″ × 2″, blue/gray) records the transmitted spectrum. Source–sample and sample–detector distances are 15 cm and 5 cm, respectively.

Photon transport simulations were performed using GEANT4 (version 10.7.3), which enables explicit control over material composition, source definition, physics processes, and detector geometry [28]. The  electromagnetic physics list G4EmStandardPhysics_option4 was used to provide accurate modeling of low- and intermediate-energy photon interactions. A narrow-beam transmission configuration was implemented in which a monoenergetic γ source (15 keV–15 MeV) irradiated an HDPE–zeolite slab placed 15 cm downstream, while a virtual scoring plane representing a NaI(Tl) detector was positioned 5 cm behind the slab to record transmitted photons. The GEANT4 attenuation geometry used in this work is illustrated in Figure 1.

All relevant EM processes were enabled, including the photoelectric effect, Compton scattering, Rayleigh scattering, electron/positron transport, and pair production at high energies. Each simulation was run with $1\times 10^{6}$ primary photons, yielding statistical uncertainties below 1–2% in the transmitted fluence. For each composite material, the linear attenuation coefficient μ was obtained using

(8) $$\mu =-\frac{1}{x}\ln\left(\frac{I}{I_{0}}\right),$$

where $I_{0}$ and *I* denote the number of photons incident on and transmitted through the slab, respectively. Composite materials were defined with the element-wise mass fractions and mixture-rule densities listed in Table 2, treating each formulation as a homogeneous medium. To ensure consistency, geometry and source–detector distances were kept constant across all compositions. GEANT4-derived μ values were compared with Phy-X/PSD outputs over the common energy grid, providing an internal cross-check of both transport physics and material definitions.

## 5. Results and Discussion

This section presents the photon attenuation characteristics of HDPE- and PLA-based composites reinforced with zeolite at different loadings (0, 10, 20, 30, and 40 wt%). The key parameters include the linear attenuation coefficient (μ), mass attenuation coefficient (μ/ρ), half-value layer (HVL), mean free path (λ), and effective atomic number ($Z_{\mathrm{eff}}$), evaluated across photon energies from 15 keV to 15 MeV using both Phy-X/PSD and GEANT4 simulations. The comparison between the two computational approaches revealed excellent agreement, with discrepancies typically below 2%, validating both the physics models and geometry configurations used.

### 5.1. Gamma Shielding Properties of HDPE–Zeolite Composites

**Figure 2:**
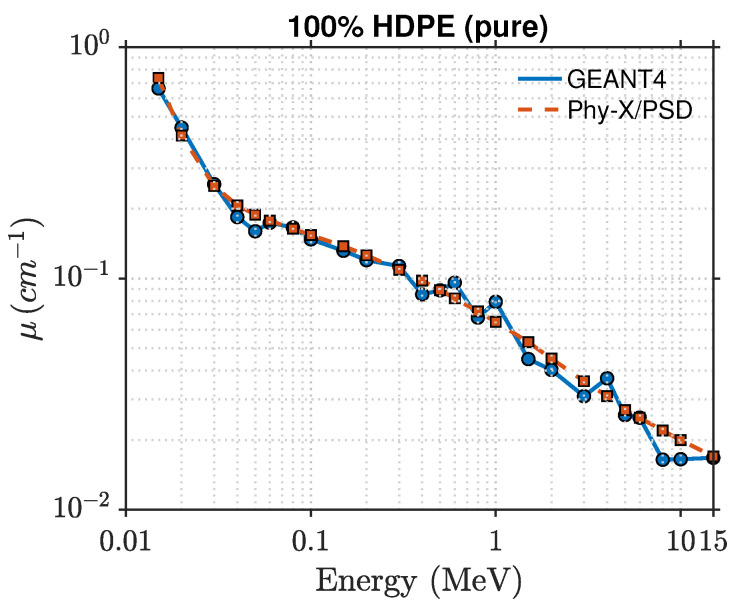
Photon attenuation comparison for 100% HDPE obtained from GEANT4 and Phy-X/PSD.

**Figure 3:**
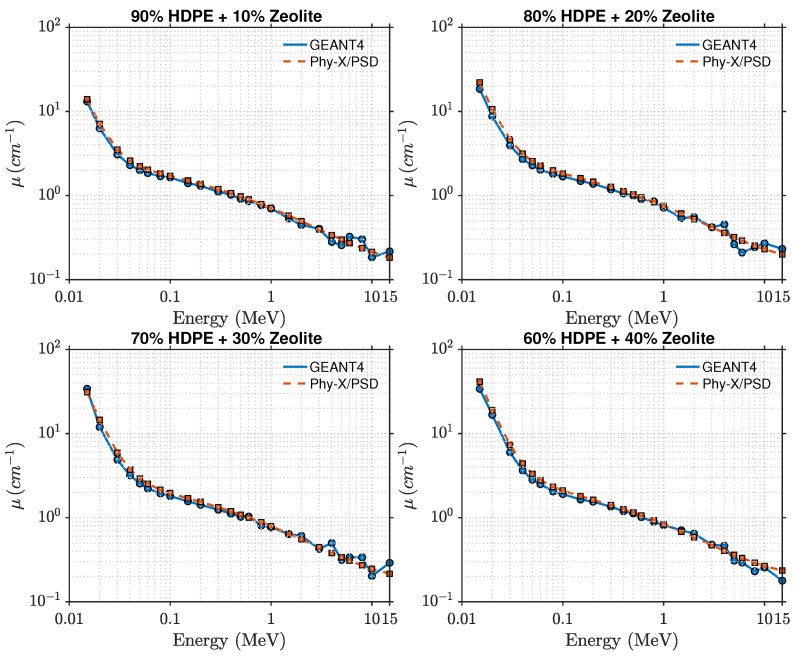
Comparison of GEANT4 and Phy-X/PSD attenuation coefficients for HDPE–zeolite composites at 10, 20, 30, and 40 wt%.

The photon attenuation behavior of pure HDPE and HDPE–zeolite composites is shown in Figure 2 and Figure 3. As expected, the linear attenuation coefficient (μ) decreases with increasing photon energy, reflecting the transition from photoelectric absorption at low energies to Compton scattering in the intermediate range. At 15 keV, μ increases from approximately $0.74\;{\mathrm{cm}}^{-1}$ for pure HDPE to about $41.5\;{\mathrm{cm}}^{-1}$ for the 40 wt% zeolite composite, indicating a strong enhancement in photon absorption. The intermediate compositions exhibit a consistent trend, confirming the progressive improvement in attenuation with higher zeolite loading. When the photon energy exceeds about 0.1 MeV, μ decreases rapidly for all compositions. In the MeV region, the attenuation curves converge, consistent with the dominance of Compton scattering, though slight composition-dependent differences remain. For instance, at 1 MeV, the 40 wt% composite maintains a marginally higher μ value than pure HDPE, while above 10 MeV, pair production begins to contribute slightly.

**Figure 4:**
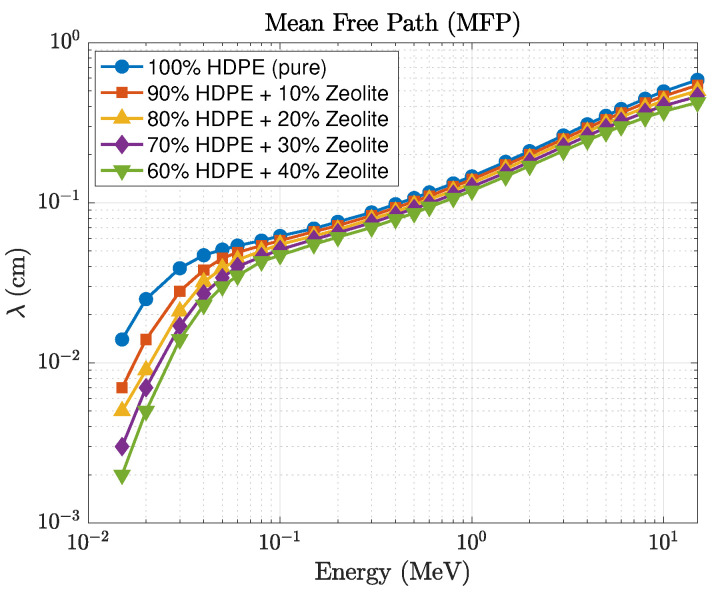
Energy dependence of the mean free path (λ) for HDPE–zeolite composites.

**Figure 5:**
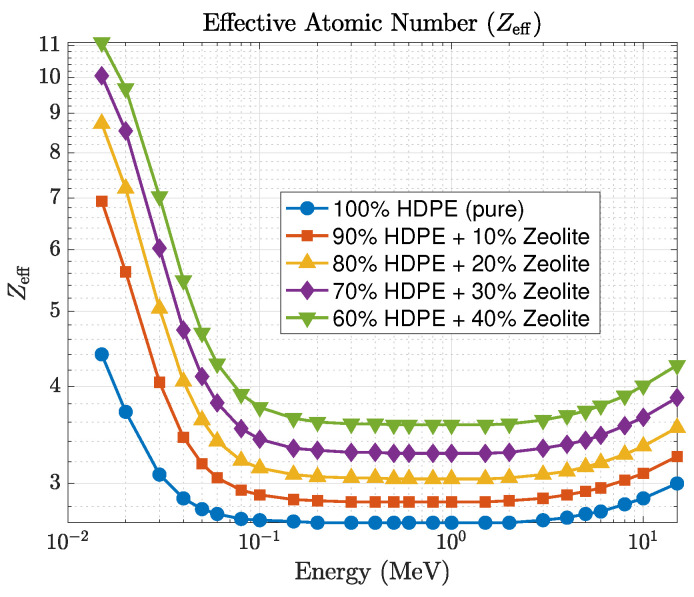
Effective atomic number ($Z_{\mathrm{eff}}$) variation with photon energy for HDPE–zeolite composites.

The comparison between the GEANT4-simulated and Phy-X/PSD-calculated values of the linear attenuation coefficient (μ) shows very close agreement, with relative deviations remaining within about 2% across all photon energies and compositions. This consistency confirms that the photon cross-section data implemented in Phy-X/PSD are well reproduced by the transport physics models in GEANT4. It also indicates that the material definitions and energy-sampling procedures used in the simulations were accurately established. The observed improvement in shielding performance is directly linked to the increase in μ, while the corresponding mean free path decreases proportionally, as illustrated in Figure 4. At 0.1 MeV, for example, pure HDPE exhibits $\mu \approx 0.062\;{\mathrm{cm}}^{-1}$, corresponding to λ≈16.1 cm and HVL≈11.2 cm, while the 40 wt% composite achieves $\mu \approx 0.21\;{\mathrm{cm}}^{-1}$, λ≈4.7 cm, and HVL≈3.3 cm. These values clearly demonstrate that higher zeolite concentrations lead to shorter penetration depths and better photon attenuation. The effective atomic number ($Z_{\mathrm{eff}}$), presented in Figure 5, also follows the expected dependence on energy and composition. For pure HDPE, $Z_{\mathrm{eff}}$ remains nearly constant around 2.7, increasing gradually to about 3.8 for the 40 wt% composite. This increase directly contributes to the enhanced low-energy absorption, where photoelectric interactions are highly sensitive to atomic number.

### 5.2. Gamma Shielding Properties of PLA–Zeolite Composites

**Figure 6:**
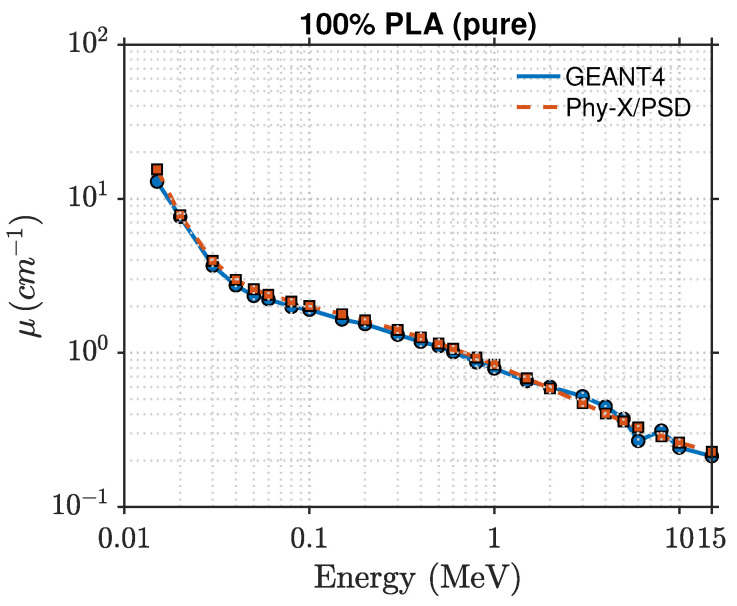
Photon attenuation comparison for 100%PLA obtained from GEANT4 and Phy-X/PSD.

**Figure 7:**
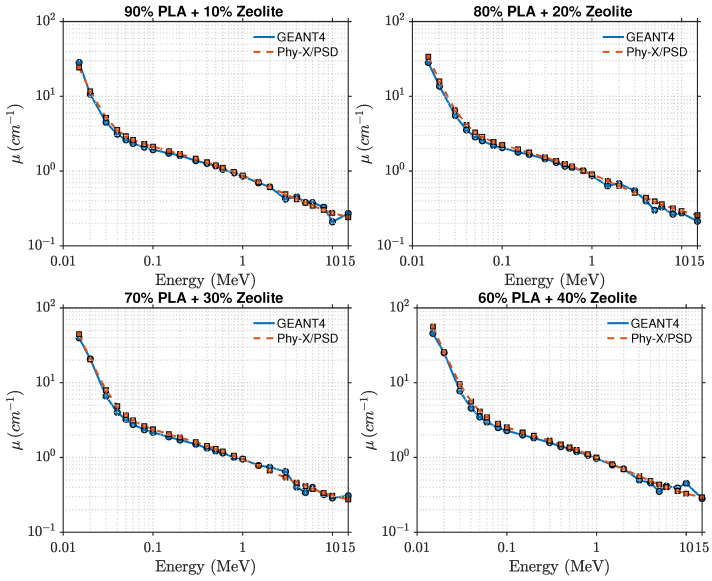
Comparison of GEANT4 and Phy-X/PSD attenuation coefficients for PLA–zeolite composites at 10, 20, 30, and 40 wt%.

Figure 6 and Figure 7 illustrate the photon attenuation characteristics of the PLA–zeolite composites. Because of its higher intrinsic density (1.25 g cm^−3^) and oxygen-rich molecular structure, PLA shows larger attenuation coefficients than HDPE across the examined energy range.

At 15 keV, the linear attenuation coefficient (μ) increases from roughly $15.5\;{\mathrm{cm}}^{-1}$ for pure PLA to about $56\;{\mathrm{cm}}^{-1}$ for the 40 wt% composite, revealing a clear improvement in low-energy photon absorption as the zeolite content increases. As the photon energy increases, the linear attenuation coefficient (μ) drops rapidly. This behavior arises from the reduced contribution of the photoelectric effect and the growing dominance of Compton scattering above approximately 0.1 MeV. Beyond 1 MeV, the attenuation curves for all compositions begin to overlap, indicating that attenuation in this energy range becomes largely independent of composition.

**Figure 8:**
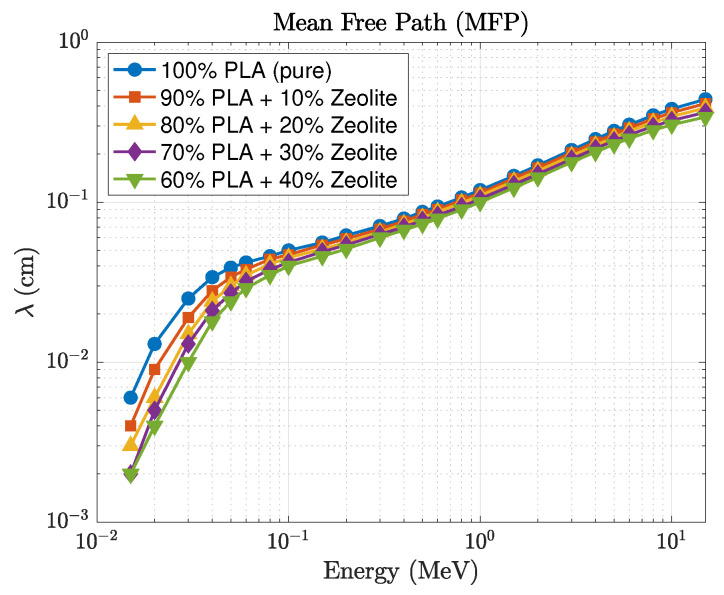
Energy dependence of the mean free path (λ) for PLA–zeolite composites.

**Figure 9:**
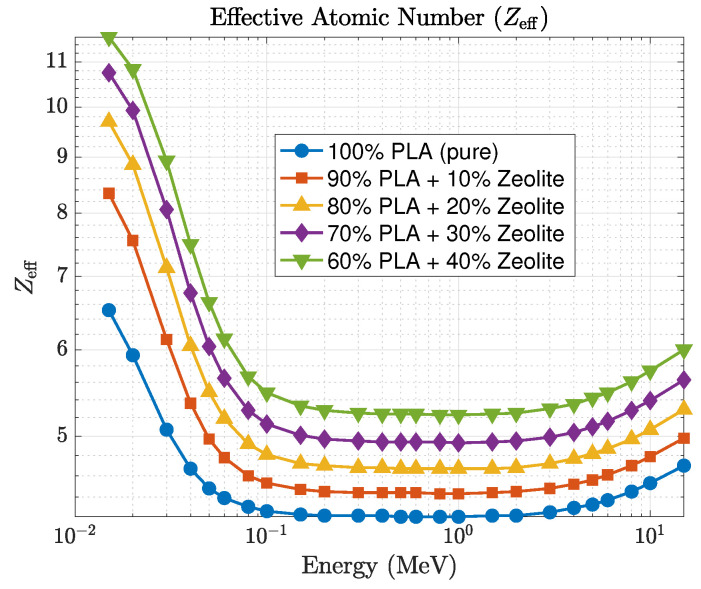
Effective atomic number ($Z_{\mathrm{eff}}$) variation with photon energy for PLA–zeolite composites.

A comparison between the GEANT4 and Phy-X/PSD datasets shows strong consistency, with relative deviations remaining within about 2% across the full energy range and all filler concentrations. This agreement highlights the reliability of both computational approaches and the accuracy of the material definitions employed in the simulations. The mean free path, shown in Figure 8, and half-value layer decrease systematically with increasing zeolite loading, consistent with the enhanced linear attenuation coefficient. This trend confirms that higher zeolite concentrations reduce photon penetration depth and improve shielding performance. The effective atomic number, shown in Figure 9, varies systematically with both photon energy and composition. At lower photon energies (15–50 keV), $Z_{\mathrm{eff}}$ rises from about 6.5 for pure PLA to nearly 11.6 for the 40 wt% composite. This increase reflects the presence of higher atomic number elements such as silicon, aluminum, and iron in the zeolite framework. When the photon energy exceeds roughly 0.3 MeV, $Z_{\mathrm{eff}}$ stabilizes around 5–6, a region dominated by Compton scattering where attenuation becomes largely insensitive to atomic number. These findings indicate that PLA–zeolite composites provide stronger photon attenuation than their HDPE-based counterparts, primarily due to their greater density and higher effective atomic number. These attributes make them excellent candidates for lightweight, durable, and environmentally friendly radiation-shielding applications in both structural and protective components.

The increase in attenuation with higher zeolite loading can be traced directly to the mineral’s oxide composition. Small amounts of Fe_2_O_3_ (Fe) and CaO (Ca) play an outsized role in raising the effective atomic number, especially at low photon energies where the photoelectric effect dominates. The major oxides SiO_2_ and Al_2_O_3_ mainly contribute by increasing the overall density and providing a moderate boost to μ. Meanwhile, alkali oxides such as K_2_O and Na_2_O add to the low-energy response because of their relatively higher atomic numbers. Taken together, this composition explains the upward trend in μ and $Z_{\mathrm{eff}}$ with increasing zeolite content, showing that the improvement arises not only from higher density but also from the specific elemental makeup of the zeolite.

### 5.3. Practical Considerations for Composite Fabrication

In practical processing of zeolite–polymer composites, several material and manufacturing considerations accompany the improvements in photon attenuation. First, uniform dispersion of the zeolite is required to avoid particle agglomeration, which can reduce mechanical strength; previous studies show that micron-scale zeolite powders can be homogeneously mixed into polymers up to 30–40 wt% using melt compounding or twin-screw extrusion [19,26]. At 40 wt% loading, however, a noticeable increase in melt viscosity is expected, particularly for HDPE, which may require higher processing temperatures or reduced molding speeds. PLA, by contrast, is more susceptible to thermal and hydrolytic degradation during extended residence times, and processing conditions must therefore be carefully controlled.

High inorganic loadings also influence mechanical behavior. Previous studies on mineral-filled polymers reports that stiffness generally increases while elongation at break and impact resistance decrease at filler contents above 20–30 wt% [17]. For this reason, the HDPE– and PLA–zeolite systems investigated here are used as model matrices for benchmarking photon attenuation rather than as optimized structural materials. In practical, long-term shielding applications, the same zeolite formulations could be transferred to radiation-resistant engineering polymers such as polyimides [12,13], which better withstand accumulated dose and thermal cycling.

### 5.4. Comparative Assessment

The results show that both HDPE– and PLA–zeolite composites exhibit a clear and progressive enhancement in photon attenuation with increasing zeolite concentration. The addition of zeolite increases the linear attenuation coefficient, reduces the mean free path, and raises the effective atomic number, reflecting the influence of high-*Z* oxide constituents that strengthen photon interactions, particularly at low and intermediate photon energies. Between the two polymer matrices, PLA-based composites consistently achieve higher values of μ and $Z_{\mathrm{eff}}$. This improvement arises from PLA’s higher intrinsic density (1.25 g cm^−3^) and its oxygen-rich molecular structure, both of which promote stronger photoelectric absorption. HDPE-based composites show slightly lower photon attenuation but remain attractive for applications where mechanical flexibility, low weight, and ease of processing are required. Accordingly, PLA–zeolite systems are well suited for applications requiring enhanced γ-ray attenuation, while HDPE–zeolite composites may be preferred in situations that prioritize structural flexibility and low mass. The close agreement between the GEANT4 and Phy-X/PSD datasets across all energies and compositions further confirms the reliability of both computational frameworks. These findings provide a solid basis for the continued development of lightweight, lead-free radiation-shielding materials for medical imaging, nuclear-safety infrastructure, and aerospace technologies.

### 5.5. Comparison with Conventional Shielding Materials

To place the simulated HDPE–zeolite and PLA–zeolite composites in context, their attenuation performance was compared with representative values for lead, structural steel, and ordinary Portland concrete at 662 keV, using tabulated NIST XCOM data and literature values reported in earlier studies [1,2,3,19,20,22]. Table 3 summarizes the typical linear attenuation coefficients and half-value layers (HVLs) for these reference materials. As expected, lead exhibits the highest attenuation ($\mu \approx 1.24\;{\mathrm{cm}}^{-1}$), followed by structural steel ($\mu \approx 0.51\;{\mathrm{cm}}^{-1}$) and Portland concrete ($\mu \approx 0.23\;{\mathrm{cm}}^{-1}$), which is consistent with previously reported measurements for standard radiation-shielding materials [1,2,22].

In comparison, the PLA–zeolite systems in this study achieve $\mu =0.16\;{\mathrm{cm}}^{-1}$ at 40 wt% loading, while the HDPE–zeolite systems reach $\mu =0.13\;{\mathrm{cm}}^{-1}$, which is consistent with the lower effective atomic numbers of polymer-based composites. Although the zeolite–polymer composites do not match the attenuation performance of high-density metals, their HVLs (4.3–5.3 cm) are comparable to the range reported for lightweight concretes, polymer–cement mixtures, and zeolite–concrete composites [1,19,20]. These results highlight the potential value of such composites in applications where low mass, lead-free composition, ease of fabrication, or environmental considerations are prioritized over maximum attenuation efficiency.

This comparison indicates that zeolite–polymer composites are not intended to replace lead or high-density concrete in high-flux environments but rather to complement existing shielding systems in scenarios requiring a balance between attenuation efficiency, mass, manufacturability, and environmental sustainability. This positioning is consistent with earlier studies on zeolite- and polymer-based shielding materials [19,20,26] and highlights the niche where the proposed HDPE– and PLA–zeolite formulations can offer practical advantages.

## 6. Conclusions

This study comprehensively evaluated the photon attenuation characteristics of HDPE- and PLA-based composites reinforced with zeolite over the energy range of 15 keV–15 MeV using Phy-X/PSD calculations and GEANT4 Monte Carlo simulations. This work serves as a feasibility and benchmarking study, and long-term structural shielding applications will require transferring the same zeolite formulations to radiation-resistant polymers rather than HDPE or PLA. Excellent consistency between the two approaches, with deviations below 2%, confirmed the reliability of both the theoretical and transport-simulation methods. The linear attenuation coefficient (μ) decreased with increasing photon energy, whereas the mean free path (λ) increased, exhibiting the typical transition from photoelectric absorption at low energies to Compton scattering at intermediate energies. Incorporation of zeolite up to 40 wt% significantly enhanced attenuation capability for both matrices owing to the increased density and effective atomic number of the composites. At 15 keV, the half-value layer (HVL) decreased from 0.60 cm to 0.08 cm for HDPE and from 0.043 cm to 0.033 cm for PLA, reflecting the stronger photon-shielding performance of the PLA–zeolite system. The effective atomic number ($Z_{\mathrm{eff}}$) increased from 2.7 to 4.3 for HDPE and from 6.5 to 11.6 for PLA with increasing zeolite loading, confirming the role of zeolite’s high-*Z* oxides in strengthening photon interactions. Between the two polymer matrices, PLA–zeolite composites exhibited superior photon attenuation across all energies, whereas HDPE–zeolite materials remain attractive for applications that prioritize low weight and ease of processing. Zeolite–polymer composites therefore represent lightweight, environmentally safe, and lead-free materials with promising potential for medical imaging, nuclear safety, and radiation-protection applications where efficient photon shielding is required. 

## Figures and Tables

**Table 1:** Oxide composition of the natural clinoptilolite zeolite used in this study (mass fractions in wt%), based on refs. [19,20].

Oxide	Mass Fraction (wt%)
SiO_2_	55.29
Al_2_O_3_	12.63
CaO	4.69
Fe_2_O_3_	3.43
MgO	1.38
Na_2_O	0.82
K_2_O	2.03
TiO_2_	0.36
Loss on Ignition (LOI)	14.71

**Table 2:** Densities of HDPE–zeolite and PLA–zeolite composites at different filler loadings (10–40 wt%).

Composite	Density (g cm^−3^)
HDPE (100 wt%)	0.940
PLA (100 wt%)	1.250
Zeolite (100 wt%)	2.200
HDPE + 10 wt% zeolite	0.9971
HDPE + 20 wt% zeolite	1.0616
HDPE + 30 wt% zeolite	1.1350
HDPE + 40 wt% zeolite	1.2193
PLA + 10 wt% zeolite	1.3064
PLA + 20 wt% zeolite	1.3682
PLA + 30 wt% zeolite	1.4360
PLA + 40 wt% zeolite	1.5110

**Table 3:** Representative linear attenuation coefficients and half-value layers (HVLs) of common shielding materials at 662 keV. Values for HDPE–zeolite and PLA–zeolite correspond to the 40 wt% composites.

Material	μ (cm^−1^)	HVL (cm)
Lead	1.24	0.56
Structural steel	0.51	1.36
Ordinary Portland concrete	0.23	3.01
HDPE–zeolite (40 wt%)	0.13	5.3
PLA–zeolite (40 wt%)	0.16	4.3

## Data Availability

The original contributions presented in this study are included in the article. Further inquiries can be directed to the corresponding author.
